# Nursing patient record practice and associated factors among nurses working in North Shewa Zone public hospitals, Ethiopia

**DOI:** 10.3389/frhs.2024.1340252

**Published:** 2024-02-08

**Authors:** Mesfin Tadese, Agizew Endale, Wondwosen Asegidew, Saba Desta Tessema, Wondimeneh Shibabaw Shiferaw

**Affiliations:** ^1^ Department of Midwifery, School of Nursing and Midwifery, Asrat Woldeyes Health Science Campus, Debre Berhan University, Debre Berhan, Ethiopia; ^2^ Department of Nursing, Debre Berhan Health Science College, Debre Berhan, Ethiopia; ^3^ Department of Public Health, Asrat Woldeyes Health Science Campus, Debre Berhan University, Debre Berhan, Ethiopia; ^4^ Department of Nursing, School of Nursing and Midwifery, Asrat Woldeyes Health Science Campus, Debre Berhan University, Debre Berhan, Ethiopia; ^5^ School of Public Health, Faculty of Medicine, The University of Queensland, Brisbane, QLD, Australia

**Keywords:** nursing documentation, factors, nurses, hospital, Ethiopia

## Abstract

**Background:**

Nursing documentation is an essential component of nursing practice and has the potential to improve patient care outcomes. Poor documentation of nursing care activities among nurses has been shown to have negative impacts on healthcare quality.

**Objective:**

To assess the nursing documentation practice and its associated factors among nurses working in the North Shewa Zone public hospitals, Ethiopia.

**Method:**

An institution-based cross-sectional study was conducted at the North Shewa Zone public hospitals. A simple random sampling technique was used to select 421 nurses. A pretested, structured, self-administered questionnaire was used to gather the data. Data were entered into Epi Data version 3.1, and SPSS version 25 was used for further analysis. Binary logistic regressions were performed to identify the independent predictors of nursing documentation practice. Adjusted odds ratio was calculated and a *p*-value less than 0.05 with 95% confidence interval (CI) was considered as statistically significant.

**Result:**

A total of 421 respondents took part, giving the survey a 100% response rate. The overall good practice of nursing care documentation was 51.1%, 95% CI (46.6, 55.8). In addition, 43.2%, 95% CI (38.5, 48.0) and 35.6%, 95% CI (30.9, 40.1), of nurses had good knowledge of and favorable attitudes toward nursing care documentation. Availability of operational standards for nursing documentation [adjusted odds ratio (AOR) = 1.76; 95% CI: 1.18, 2.64], availability of documenting sheets (AOR = 1.51; 95% CI: 1.01, 2.29), and a monitoring system (AOR = 1.61; 95% CI: 1.07, 2.41) were significantly associated with nursing care documentation practice.

**Conclusion:**

Nearly half of nursing care was not documented. The practice of nursing care documentation was significantly influenced by the availability of operational standards, documenting sheets, and monitoring systems. To improve the documentation practice, a continuous monitoring system and access to operational standards and documenting sheets are needed.

## Background

Nursing documentation is a record of the nursing care that was planned for and provided to specific patients by licensed nurses or other caregivers acting under a licensed nurse's guidance (1). It accurately reflects nurse evaluations, changes in clinical status, treatment given, and pertinent patient information. Documentation is the primary clinical information source to fulfill legal and professional standards (2). It is a crucial part of the nursing process as it is a vital form of communication within the healthcare team regarding patient care. Nursing care documentation helps the multidisciplinary care team identify patients’ deteriorating conditions earlier and respond to them effectively (3).

Even though documentation confirms the quality and effectiveness of nursing care, most nurses have poor documentation practices. According to a study on the nurses in the Papuan province of Indonesia, only 37% of them had good documentation practices (4). Similarly, in Ghana, 46% of the patient care was not documented in nursing care records, 63% of the patients did not get progress notes from the nurses after the first day of admission, and 57% of the documentation was not signed by the nurses (5). Nursing care documentation was also inadequate in the Southern (42%) (2) and Northwestern Ethiopia (37.4%) (6).

Inadequate documentation among nurses has been demonstrated to negatively affect patients’ outcomes, treatment decisions, diagnostic methods, the performance of nurses, and the profession as a whole. It is also linked to the issue of omitting prescriptions, which increases the risk of legal harm that involves severe injury or death of a patient (6, 7). A study in Bahir Dar, Northwest Ethiopia, revealed that 87.5% of medication administrations have erroneous documentation (8). Nursing records are the only proof that a patient's treatment commitment was fulfilled when they filed a complaint. Thus, focusing on nursing documentation will safeguard its legal implications.

A World Health Organization (WHO) survey revealed that one contributing factor of medical errors is inadequate communication among healthcare providers (9). In addition, there is evidence to suggest a connection between patient mortality and maintaining nurse records (10). Despite the fact that maintaining a patient record is required of nurses by law, numerous studies have revealed shortcomings in documenting practices among nurses worldwide (11). It has been noted that the nursing records are frequently inaccurate, fragmentary, and of low quality (12–14).

Several researches recommended utilizing a multidisciplinary approach to solve documentation issues. These include developing rules and regulations (1, 7) and providing continuing opportunities for nurses to receive training on the effectiveness of documentation (2, 6, 15). It is also expected of nursing leaders to encourage, support, and hire more personnel (6, 15).

Several factors determine how closely nurses are adhering to the nursing care documentation procedure. This includes age, educational status, operational standard of nursing documentation, hospital level, in-service training on standard nursing processes, work experience (>5 years), and availability of documenting sheets (1, 2). Demotivation, workload, shortage of time, and inadequate administrative assistance are also factors that contribute to poor patient record-keeping quality (16). In addition, there is a strong relationship between nursing documentation and low nurse-to-patient ratio, knowledge, and attitude toward documentation practice (6).

Insufficient nursing documentation is a significant challenge for the delivery of quality nursing healthcare services. Although there has been some research on nursing documentation conducted in Ethiopia, issues like fatigue, a lack of promotion, and inadequate monitoring and evaluation have not been adequately addressed. The results of the study will be valuable to nurses as they advance their knowledge of and attitudes toward documentation, which will improve care coordination and client safety. Thus, the study aimed to assess the nursing documentation practice and its associated factors in North Shewa Zone public hospitals, in Ethiopia.

## Methods and materials

### Study design, setting, and period

This cross-sectional study was conducted in the North Shewa Zone public hospitals, in Ethiopia, from 2 April 2021 to 9 May 2021. North Shewa is one of the 12 zones in the Amhara regional state. Debre Berhan Town, which is 130 km from Addis Ababa and 695 km from Bahir Dar, the capital of the Amhara Regional State, serves as the city's administrative center. The overall population of the zone is 2,299,203. There are 12 hospitals (2 private and 10 public), 24 health centers, and 17 private clinics overall in the zone. There are 766 nurses working in the area with diplomas, bachelor's degrees, and master's degrees.

### Study population and inclusion criteria

All nurses working in North Shewa Zone governmental hospitals were the study population. Included were professional nurses who gave direct patient care, had at least 6 months of work experience, and agreed to participate voluntarily. Nurses who were unable to participate due to illness, had less than 6 months of experience, and who were on maternity or annual leave at the time of data collection were excluded.

### Sample size determination

The sample size required for the study was calculated using Open-Epi statistical software, version 3.03. A 95% confidence interval (CI), a 5% margin of error, and a 47.5% prevalence of nursing documentation practice (15) were the assumptions made. Adding a 10% non-response rate, the estimated sample size was 421.

### Sampling procedure

A cluster random sampling method was used to recruit the samples. A simple random sampling technique was used to select the hospitals. Six hospitals were chosen by lottery out of the 10 public hospitals in the zone; they are the Debre Berhan Comprehensive Specialized Hospital (DBCSH), Ataye Primary Hospital (APH), Mehal Meda General Hospital (MMGH), Shewa Robit Primary Hospital (SPH), Debre Sina Primary Hospital (DSPH), and Deneba Primary Hospital (DPH). These six hospitals have 330, 42, 44, 30, 26, and 26 nurses, respectively. The list of all nurses working in the in-patient and out-patient departments was obtained from the chief executive officer (CEO) of the respective hospitals. The required number of nurses was obtained by proportional allocation of the sample size to each hospital based on their respective number of nurses; 279, 36, 37, 25, 22, and 22, from DBCSH, APH, MMGH, SPH, DSPH, and DPH, respectively. Finally, a computer-generated (Random Draw) simple random sampling technique was used to select the nurses from the sampling frame. After that, the data collectors contacted the target nurses to provide the questionnaire.

### Study variable

The dependent variable of the study is nursing care documentation practice. Sociodemographic factors (age, sex, educational status, year of experience, marital status, salary), organizational factors (number of staff, lack of time, fatigue, availability of operational standards, motivation from supervision, in-service training, promotion, availability of documenting sheets, familiarity with operational standard, working environment, and lack of monitoring and evaluation), and knowledge of and attitudes toward nursing documentation are among the explanatory variables that can be used to explain the data.

### Definition of terms

#### Nursing documentation practice

This was measured using 18 questions. A value of 3, 2, 1, and 0 were scored for the options “Always,” “Sometimes,” “Rarely,” and “Never,” respectively. The participant's performance was categorized into Good Practice when scored above or equal to the mean score of the practice questions, and Poor Practice when scored below the mean value (1, 2, 15).

#### Knowledge of nursing documentation

This was measured using 10 items, with “Yes,” “No,” and “I don't know” as the response options. The correct response received a score of 1, while the incorrect responses received a score of 0. Those who scored greater than or equal to the mean score of correct answers were categorized as having good knowledge and those who scored less than the mean value were categorized as having poor knowledge of nursing care documentation (1, 2, 6).

#### Attitudes toward nursing documentation

These were assessed using nine items with a three-point Likert scale, with a score of two for “Agree,” 1 for “Neutral,” and 0 for “Disagree.” The participants were classified as having favorable attitudes if their scores on all of the attitude questions were greater than or equal to the mean score, and they were classified as having unfavorable attitudes if their scores fell below the mean value (1, 2, 6).

### Data collection tool and procedure

Data were collected using a pretested, structured, self-administered English version questionnaire developed from previous similar studies (1, 2, 6, 11, 17, 18). There are five sections in the questionnaire, addressing the following: (1) sociodemographic background; (2) organizational characteristics; (3) the practice of nursing documentation; (4) nNurses’ knowledge of documentation; and (5) attitudes toward nursing documentation (Supplementary Material S1). Data collection involved six research nurses with BSc degrees (five data collectors and one supervisor), none of whom were employees of the study hospitals. The objective, methodology, requirement for information confidentiality, and data collection techniques were all covered during a 1-day training session in the town of Debre Berhan.

### Data quality assurance

To maintain the quality of the data, a standardized individual nursing documentation practice tool developed by the Federal Democratic Republic of Ethiopian Ministry of Health was used (18). Before the actual data collection, a pretest was done on 5% (21 nurses) of the sample in Enat Hospital. Cronbach's alpha was evaluated for item internal consistency, and the values for the knowledge, attitude, and practice questions were 0.71, 0.83, and 0.74, respectively. The content validity of the tool was evaluated by a senior quality team expert. Regular supervision, short and clear questions, and the avoidance of leading questions all were followed to reduce the non-response rate.

### Data processing and analysis

Data were cleaned, edited, and entered into the EPI data version 3.1 and moved to the SPSS version 25 statistical software for further analysis. Descriptive statistics were used to organize and summarize the variables. Bivariable analysis for each independent variable with the outcome variable was performed to select the candidates for the multivariable analysis. All variables with *p*-value <0.25 on the bivariable analysis were taken for the multivariable analysis. Multicollinearity test was run among independent variables and there was no multicollinearity. The Hosmer‒Lemeshow goodness-of-fit test was done and the model was fitted. The adjusted odd ratio was calculated and a *p*-value less than 0.05 with 95% CI was taken as statistically significant. Finally, the results were presented using texts, tables, and figures.

### Ethical approval

Ethical clearance was obtained from the institutional review board of the Debre Berhan University with institutional research ethics review committee number of IRB protocol: DBU/CHS/MN/SG11/2020. A support letter was written to each hospital. The purpose of the study was clearly explained to the participants and their written informed consent was obtained before data collection (Supplementary Material S2). The study was conducted in accordance with the Declaration of Helsinki ethical principles for medical research on human subjects.

## Results

### Sociodemographic characteristics of participants

A total of 421 respondents took part, giving the survey a 100% response rate. The participants had a mean (SD) age of 30.44 ± 5.362 and ranged from 21 to 50 years. Over half, 54.9%, of them were women. Three-fourth (74.6%) of participants had a BSc degree and 56.3% had no more than 5 years of professional nursing experience. More than half (54.9%) of the nurses were married and 59.4% of them earned a monthly income of 5,001–7,071 Ethiopian birrs (Table 1).

**Table 1:** Sociodemographic characteristics of nurses in public hospitals of North Shewa Zone, Ethiopia, 2021.

Variables	Category	Frequency	Percent (%)
Age (years)	21–25	78	18.5
	26–30	180	42.8
	31–35	70	16.6
	≥36	93	22.1
Sex	Male	190	45.1
	Female	231	54.9
Level of education	College diploma	90	21.4
	Bachelor’s degree	310	73.6
	MSc degree	21	5.0
Marital status	Single	165	39.2
	Married	231	54.9
	Divorced	25	5.9
Work experience in years	≤5	237	56.3
	6–10	146	34.7
	11–15	20	4.8
	≥16	18	4.3
Monthly income in Ethiopian Birr	3,000–5,000	78	18.5
	5,001–7,071	250	59.4
	7,072–9,000	60	14.3
	>9,000	33	7.8

### Organizational related factors

Nearly half (45.8%) of the participants received in-service training on standard nursing care documentation. Of them, 61.6% of the nurses received in-service training within the past 2 years. Approximately 238 (56.5%) of the nurses do not have adequate time to record their nursing activities after providing care. Moreover, half of the respondents (58%) were familiar with the operational standards of nursing documentation, and 59.4% had access to nursing care plan sheets in their workplace (Table 2).

**Table 2:** Frequency distribution of organizational factors among nurses working in North Shewa Zone public hospitals, Ethiopia, 2021.

Variables	Category	Frequency	Percent (%)
Working unit	OPD	180	42.8
	IPD	241	57.2
In-service training	Yes	193	45.8
	No	228	54.2
Time received the training (*n* = 193)	≤2 years ago	114	59.0
	3–5 years ago	70	36.3
	≥6 years ago	9	4.7
Shortage of time for documentation	Yes	238	56.5
	No	183	43.5
Availability of operational standard for documentation	Yes	233	55.3
	No	188	44.7
Familiarity with the standard	Yes	244	58.0
	No	177	42.0
Adequate nursing care plan sheet	Yes	250	59.4
	No	171	40.6
Motivation from a supervisor	Yes	193	45.8
	No	228	54.2
Type of motivation (*n* = 193)	Oral appreciation	120	62.2
	Certificate	57	29.5
	Salary increment	16	8.3
Availability of promotion from hospital	Yes	231	54.9
	No	190	45.1
Adequacy of staff	Yes	187	44.4
	No	234	55.6
Feel fatigued while documentation	Yes	239	56.8
	No	182	43.2
Availability of monitoring system	Yes	249	59.1
	No	172	40.9
Availability of evaluation system	Yes	233	55.3
	No	188	44.7

IPD, in-patient department; OPD, out-patient department.

### Knowledge of nursing documentation

The mean (±SD) cumulative score of knowledge questions was 6.27 ± 1.83. The overall prevalence of nurses with a good knowledge of nursing documentation was 43.2% (*N* = 182), 95% CI (38.5, 48.0). In addition, 69.1% of the nurses know that documentation is part of their professional responsibility, 61.8% are aware that complete documentation is a principle that must be followed while documenting, and 67.7% understand that regular documentation can protect them from legal suits (Table 3).

**Table 3:** Nurse's knowledge of documentation in North Shewa public hospitals, Ethiopia, 2021.

Statements	Yes (%), *n* (%)	No (%), *n* (%)
Documentation is part of the professional responsibility	291 (69.1)	130 (30.9)
Complete documentation is a principle need of documentation	260 (61.8)	161 (38.2)
Easily readable nursing documentation is a principle	320 (76.0)	101 (24.0)
Error-free nursing documentation is a principle for documentation	246 (58.4)	175 (41.6)
Improved quality of care is an advantage of patient care documentation	294 (69.8)	127 (30.2)
Patient care documentation is important for better communication	266 (63.2)	155 (36.8)
Legal protection is an advantage of patient care documentation	288 (68.4)	133 (31.6)
Inadequate documentation has a potential consequence	240 (57.0)	181 (43.0
Recording only what you saw is important	249 (59.1)	172 (40.9)
Regular documentation can protect from legal suit	285 (67.7)	136 (32.3)

### Attitudes toward nursing documentation

The mean (±SD) score of attitude questions was 13.15 ± 4.56. The overall prevalence of nurses with a favorable attitude toward nursing documentation was 35.6% (*N* = 150), 95% CI (30.9, 40.1). In addition, 75% of the nurses agreed that nursing care documentation has an advantage in daily work, 73.1% indicated that proper documentation has a positive impact on patient safety, and 75.3% of the nurses accept that high-quality nursing care documentation can add value to the hospital (Table 4).

**Table 4:** Attitudes of nurses toward nursing documentation in North Shewa Zone public hospitals, Ethiopia, 2021*.*

Variables	Agree, *n* (%)	Neutral, *n* (%)	Disagree, *n* (%)
Nursing documentation has an advantage	316 (75.0)	21 (5.0)	84 (20.0)
Proper documentation has a positive impact	308 (73.1)	26 (6.2)	87 (20.7)
Nurses are expected to perform complete and accurate documentation	303 (72.0)	42 (10.0)	76 (18.0)
A well-written report can replace an oral shift report	281 (66.7)	49 (11.6)	91 (21.6)
Nursing notes are meaningful and give legal protection	320 (76.0)	45 (10.7)	56 (13.3)
Quality documentation of nursing care can add value to the hospital	317 (75.3)	55 (13.1)	49 (11.6)
Nurses have sufficient knowledge of the documentation procedure	282 (67.0)	60 (14.2)	79 (18.8)
Nursing admission assessment should be completed within 1 h	280 (66.5)	53 (12.6)	88 (20.9)
Patients participate in care planning to improve the quality of care	308 (73.2)	51 (12.1)	62 (14.7)

### Nursing care documentation practice

The mean (±SD) cumulative score of practice questions was 35.51 ± 7.87. The overall prevalence of nurses with a good practice of nursing care documentation was 51.1% (*N* = 215), 95% CI (46.6, 55.8). More than half (58.4%) of the nurses always document the care they provide for every patient, and 53.2%, 51.8%, 47.3%, 49.4%, and 44.9% of the nurses always document an assessment, diagnosis, plan, implementation, and evaluation of the patient, respectively. In addition, nearly one quarter (23.5%) of the nurses always kept the confidentiality of patient records by locking the computer with a password (Table 5).

**Table 5:** Nursing documentation practice in North Shewa Zone public hospitals, Ethiopia, 2021.

Questions	Never, *n* (%)	Rarely, *n* (%)	Sometimes, *n* (%)	Always, *n* (%)
How often do you document the care you have provided for every patient?	37 (8.8)	68 (16.2)	70 (16.6)	246 (58.4)
How often did you begin filling nursing documentation with date and time?	9 (2.1)	77 (18.3)	133 (31.6)	202 (48.0)
How often did you document an assessment for patient care?	17 (4.0)	50 (11.9)	130 (30.9)	224 (53.2)
How often did you document the diagnosis of the patient?	15 (3.6)	57 (13.5)	131 (31.1)	218 (51.8)
Have you ever documented the plan of patient care?	46 (10.9)	52 (12.4)	124 (29.4)	199 (47.3)
Have you ever documented the implementation of patient care?	39 (9.3)	62 (14.7)	112 (26.6)	208 (49.4)
How often you did document an evaluation of the patient?	23 (5.5)	62 (14.7)	147 (34.9)	189 (44.9)
How often did you end the documentation with an authorized signature?	24 (5.7)	75 (17.8)	127 (30.2)	195 (46.3)
Do you keep confidentiality of patient records, maintaining patient charts to be accessed by authorized personnel only?	55 (13.1)	64 (15.2)	102 (24.2)	200 (47.5)
Have you ever got informed consent from the client to use or disclose information to others?	56 (13.3)	66 (15.7)	138 (32.8)	161 (38.2)
How often did you maintain the confidentiality of a patient's records by a password to keep the computers safe (if electronic)?	185 (43.9)	66 (15.7)	71 (16.9)	99 (23.5)
Did you ever continue keeping the confidentiality of the patient records after the death of an individual?	48 (11.4)	51 (12.1)	113 (26.8)	209 (49.6)
How often did you read your colleagues notes?	41 (9.7)	89 (21.2)	141 (33.5)	150 (35.6)
Does your colleague's recording provide you adequate information?	52 (12.4)	116 (27.5)	134 (31.8)	119 (28.3)
How often did you document health information or advice you have provided to a patient?	54 (12.8)	95 (22.6)	122 (29.0)	150 (35.6)
Do you practice a computerized nursing care documentation system currently in your hospital?	184 (43.7)	64 (15.2)	71 (16.9)	102 (24.2)
How often did you voluntarily report any medical error that occured while providing patient care?	65 (15.4)	102 (24.3)	128 (30.4)	126 (29.9)
How often did you document the patient's response to the care you provide?	83 (19.7)	77 (18.3)	104 (24.7)	157 (37.3)

### Factors of nursing care documentation

After doing a bivariate logistic regression analysis, variables with a *p*-value of less than 0.25 were selected for the multivariable logistic regression analysis model. In the final model, the availability of operational standards, documenting sheets, and monitoring systems have shown a statistically significant association with good documentation practices.

Nurses who work in hospitals with operational standards were 76% more likely to have good documentation as compared with in hospitals without operational standards (AOR = 1.76; 95% CI: 1.18, 2.64). In comparison with nurses who did not have sufficient documenting sheets, those who did were 51% more likely to practice documentation (AOR = 1.51; 95% CI: 1.01, 2.29). Compared with hospitals without monitoring systems, nurses were 61% more likely to document nursing care in hospitals where monitoring systems were accessible for documentation (AOR = 1.61; 95% CI: 1.07, 2.41) (Table 6).

**Table 6:** Factors associated with nursing documentation practice among nurses working in North Shewa Zone public hospitals, Ethiopia, 2021.

Variables	Category	Documentation practice, *n* (%)		COR (95% CI)	AOR (95% CI)
		Poor (*n* = 206)	Good (*n* = 215)		
Time shortage	No	82 (39.8)	101 (47.0)	1.34 (0.91, 1.97)	1.39 (.93, 2.08)
	Yes	124 (60.2)	114 (53.0)	1	1
Availability of operational standard	No	110 (53.4)	78 (36.3)	1	1
	Yes	96 (46.6)	137 (63.7)	2.01 (1.36, 2.97)	1.76 (1.18, 2.64)*
Familiarity with the standard	No	98 (47.6)	79 (36.7)	1	1
	Yes	108 (52.4)	136 (63.3)	1.56 (1.06, 2.31)	1.49 (0.99, 2.24)
Availability of documenting sheet	No	97 (47.1)	74 (34.4)	1	1
	Yes	109 (52.9)	141 (65.6)	1.70 (1.15, 2.51)	1.51 (1.01, 2.29)*
Availability of monitoring system	No	97 (47.1)	75 (34.9)	1	1
	Yes	109 (52.9)	140 (65.1)	1.66 (1.12, 2.46)	1.61 (1.07, 2.41)*
Knowledge	Poor	127 (61.7)	112 (52.1)	1	1
	Good	79 (38.3)	103 (47.9)	1.48 (1.00, 2.18)	1.19 (0.79, 1.79)

* Significant at *p*-value <0.05.

## Discussion

### The practice of nursing documentation

The study's findings point out a critical hole in the thorough documentation of the nursing process that underpins and guides nurses’ clinical judgments on patient safety. The study found that 51.1%, 95% CI (46.6, 55.8), of the nurses have good documentation practices. The finding is consistent with the study done in the Tigray region (1), Harari region and Dire Dawa administration (15), West Gojjam Zone (7), and Addis Ababa (17) in which 47.8%, 47.5%, 47.5%, and 47.8% of the nurses had good documentation practices, respectively. Poor documenting of nurses’ clinical decision-making has an adverse effect on the continuity, quality, and safety of medical care, including insufficiently detecting deterioration and care escalation (19).

However, the result was higher than the 37% in Indonesia (4), 46% in Ghana (5), 37.4% in Gondar (6), and 42% in the Wolaita Zone (2). The discrepancy may be due to a difference in the study period, which may have led to information differences over time, given that the previous studies were conducted before 4 years and the current one, after technology had experienced rapid expansion, such as the introduction of smart care in the majority of Ethiopian hospitals. In addition, it could be due to the variation in the data collection technique [i.e., a previous study (15) used both self-administered questionnaires and medical record reviews], as well as evaluated nurses’ knowledge and attitudes toward documentation practices (6, 7), in-service training about documentation (17), and the possibility that nurses who took part in the current study were more familiar with standard documentation guidelines. The inclusion of different levels of hospitals in the different research studies [this study included five primary hospitals and one comprehensive specialized hospital, whereas another study (17) only used specialized hospitals] could also be a contributing factor. This may be attributed to a heavy workload, a shortage of nurses, a lack of time, and a lack of support from hospital management and leaders (1).

Conversely, the finding was lower than the reports from Jamaica 98% (11), India 67.5% (20), and Nigeria 70% (21). The potential factors that explain the variation might be differences in the socioeconomic statuses, organizational structures and setups, favorability of work environments, the number of patients served by each registered nurse, monitoring and evaluation systems, and familiarity with documentation guidelines (17).

### Factors associated with nursing care documentation practice

Nurses who have operational standards in their hospitals are more likely to document nursing care practice as compared with nurses without operational standards. This finding is in line with studies conducted in the West Gojjam Zone (7) and the Tigray region, Ethiopia (1), where the availability of operational standards had a significant association with nursing care documentation practices. This may be because standardized nursing documentation guidelines are used as a source of information about how to document nursing care given to patients. Nurses may find documentation chores to be simple, quick, and interesting if they are familiar with the operational requirements for documentation. In addition, nurses who are familiar with the operational standards are more knowledgeable, which improves their practice of documenting nursing care (6).

In comparison to nurses who did not have sufficient documentation sheets, those who did were more likely to practice documentation. This was similar to findings from the Tigray (1) and Harari regions (15), where inadequacy of documenting sheets significantly hindered documentation practices. In addition, the practice of documenting nursing care in Addis Ababa, Ethiopia, was substantially correlated with both a lack of skill and familiarity with operational standards of nursing documentation (17). The standard of healthcare services is impacted by hospital factors like the availability of supplies and equipment. The majority of nurses who took part in this study emphasized that a shortage of documenting sheets greatly restricts the quality of documentation practice.

Nurses who work in hospitals with monitoring systems are more likely to document nursing care compared with in hospitals without monitoring systems. The health sector relies heavily on monitoring and evaluation to make sure that healthcare documentations are efficient, effective, and responsive to community health needs. Nurses employed in hospitals having continuous monitoring systems were more likely to practice good documentation because of the fear of punishment and/or their desire to earn the favor of their superiors. In addition, nursing leaders are able to quickly recognize the issue with poor documentation and will resolve it.

### Limitation

Given that this survey was self-administered, social desirability bias may have overestimated or underestimated the documentation practices. Only six representative hospitals were included in the study. In addition, only nursing professionals’ records in a few public hospitals were examined and the documentation practices of nurses in private hospitals were not evaluated.

## Conclusion and recommendation

Nearly half of nursing care was not documented. The practice of nursing care documentation was significantly influenced by the availability of operational standards, documenting sheets, and monitoring systems. To improve the documentation practice, a continuous monitoring system and access to operational standards and documenting sheets are needed. It is advised to conduct further longitudinal researches to prove a cause-and-effect relationship. For more representative results, similar studies with varied study designs and measurements should be carried out in other settings. These findings will aid policymakers in the creation of interventional activities aimed at improving documentation practices.

## Data Availability

The raw data supporting the conclusions of this article will be made available by the authors, without undue reservation.
