# Supplementary material for: Nursing patient record practice and associated factors among nurses working in North Shewa Zone public hospitals, Ethiopia
Source: Front Health Serv. 2024 Feb 8;4:1340252. doi: 10.3389/frhs.2024.1340252 (PMC10883157; doi:10.3389/frhs.2024.1340252)
Supplement: Supplementary file 1 [file Table1.docx]

**Questionnaire English version**

**Section I: Socio-demographic characteristics.**

**Instruction: Please encircle the chosen option(s) or write the appropriate answer in the space provided.**

| **S. No** | **Questions** | **Response and coding** |
| --- | --- | --- |
|  | How old are you? | _________ years |
|  | Your sex? | 1. Male 2. Female. |
|  | What is the highest level of education you have attained? | 1. College diploma 2. Bachelor degree 3. MSc and above |
|  | What is your marital status? | 1. Single 2. Married 3. Divorced 4. widow/widower |
|  | Year of clinical experience | ___________ Years |
|  | Average monthly income | ___________ Ethiopian Birr |

**Section II: Organizational factors**

**Instruction: Please read the following questions carefully and encircle on the correct answer option.**

| **S. No** | **Questions** | **Response and coding** | **Skip** |
| --- | --- | --- | --- |
|  | Where is your working unit? | 1. Medical ward 2. Surgical ward 3. Pediatric ward 4. Regular OPD 5. Emergency OPD 6. Pediatric OPD 7. Gynecology ward 8. Obstetrics ward. 9. Neonatal ICU 10. Other specify________ |  |
|  | Did you get nursing standard in-service training? | 1. Yes 2. No | If No skip to Q4 |
|  | If yes for Q2, when did you get it? | ________ |  |
|  | Do you have a time shortage for documentation? | 1. Yes 2. No |  |
|  | Does your hospital have an operational standard for nursing documentation? | 1. Yes 2. No |  |
|  | Are you familiar with the operational definition? | 1. Yes 2. No |  |
|  | Does nursing care plan sheets easily available in the nursing unit? | 1. Yes 2. No |  |
|  | Does motivation from a supervisor is available in your working units? | 1. Yes 2. No | If No,  skip to Q10 |
| 9. | What type of motivation is available in your hospital? | 1. Oral appreciation 2. Certificate of recognition 3. Increase salary 4. Other specify________ |  |
| 10. | Does promotion is available in your hospital? | 1. Yes 2. No |  |
| 11. | Do you think there is an adequate number of staff for doing nursing documentations regularly? | 1. Yes 2. No |  |
| 12. | Do you ever feel fatigued while documentation? | 1. Yes 2. No |  |
| 13. | Does your hospital have a monitoring system for nursing documentation? | 1. Yes 2. No |  |
| 14. | Does your hospital have an evaluation system for nursing documentation? | 1. Yes 2. No |  |

**Section III: Knowledge of nursing documentation.**

**Instruction:** Please read the following questions carefully and encircle on the correct answer option as honestly as possible. If the statement is **correct** circle “**Yes”**, incorrect circle “**No”,** and if you are unsure circle, “**I don’t know”.**

| **S. No** | **Statement** | **Response and coding** |
| --- | --- | --- |
|  | Documentation is part of professional responsibility. | 1. Yes 2. No 3. I don’t know |
| 2. | Complete documentation is a principle needed to documentation. | 1. Yes 2. No 3. I don’t know |
| 3. | Easily readable nursing documentation is a principle. | 1. Yes 2. No 3. I don’t know |
| 4. | Error-free nursing documentation is a principle needed to be followed. | 1. Yes 2. No 3. I don’t know |
| 5. | Improved quality of care is an advantage of patient care documentation. | 1. Yes 2. No 3. I don’t know |
| 6. | Patient care documentation is important for better communication with health care staff. | 1. Yes 2. No 3. I don’t know |
| 7. | Legal protection is an advantage of patient care documentation. | 1. Yes 2. No 3. I don’t know |
| 8. | Inadequate documentation has a potential consequence for the poor development of the nursing profession. | 1. Yes 2. No 3. I don’t know |
| 9. | Recording only what you saw is important to protect you from legal suit. | 1. Yes 2. No 3. I don’t know |
| 10. | Regular documentation can protect from legal suit. | 1. Yes 2. No 3. I don’t know |

**Section IV:** **Attitude towards documentation**

**Instruction:** Please read the following statements carefully and put a **checkmark** on the option that best agrees with your opinion. Tick **“Neutral”** if you don’t want to agree nor disagree with the opinion.

| **S. No** | **Statement** | **Agree** | **Neutral** | **Disagree** |
| --- | --- | --- | --- | --- |
|  | Nursing documentation has an advantage in daily work. |  |  |  |
|  | Proper documentation has a positive impact on patient safety. |  |  |  |
|  | Nurse are expected to perform complete and accurate documentation. |  |  |  |
|  | A well-written report can replace an oral shift report. |  |  |  |
|  | Nursing notes are meaningful and give legal protection. |  |  |  |
|  | Quality documentation of nursing care can add value to the hospital. |  |  |  |
|  | Nurses have sufficient knowledge of the documentation procedure. |  |  |  |
|  | Nursing admission assessment should be completed within 1 hour. |  |  |  |
|  | Patients participate in care planning to improve the quality of care. |  |  |  |

**Section V: Nursing Documentation Practice.**

**Instruction:** Please read the following questions carefully and encircle the correct answer from the given alternatives.

| **S. No** | **Statements** | **Response and coding** |
| --- | --- | --- |
|  | How often do you document the care you have done for every Patient? | 1. Always 2. Sometimes 3. Rarely 4. Never |
|  | How often did you begin feeling nursing documentation with date and time? | 1. Always 2. Sometimes 3. Rarely 4. Never |
|  | How often did you document an assessment for patient care? | 1. Always 2. Sometimes 3. Rarely 4. Never |
|  | How often did you document the diagnosis of the patient? | 1. Always 2. Sometimes 3. Rarely 4. Never |
|  | Have you ever documented the plan of patient care? | 1. Always 2. Sometimes 3. Rarely 4. Never |
|  | Have you ever documented the implementation of patient care? | 1. Always 2. Sometimes 3. Rarely 4. Neve |
| 7. | How often you did document an evaluation of the patient? | 1. Always 2. Sometimes 3. Rarely 4. Never |
| 8. | How often did you end nursing documentation with an authorized signature? | 1. Always 2. Sometimes 3. Rarely 4. Never |
| 9. | Do you keep confidentiality of patient records maintaining patient charts to be accessed by authorized personnel only? | 1. Always 2. Sometimes 3. Rarely 4. Never |
| 10 | Have you ever got informed consent from the client to use or disclose information to others? | 1. Always 2. Sometimes 3. Rarely 4. Never |
| 11 | How often did you keep the confidentiality of patient's records by a password of computers safe (if electronic)? | 1. Always 2. Sometimes 3. Rarely 4. Never |
| 12. | Did you ever continue keeping the confidentiality of the patient records after the death of an individual? | 1. Always 2. Sometimes 3. Rarely 4. Never |
| 13. | How often did you read your colleagues ’notes? | 1. Always 2. Sometimes 3. Rarely 4. Never |
| 14. | Does your colleague’s recording provide You adequate information? | 1. Always 2. Sometimes 3. Rarely 4. Never |
| 15. | How often did you document health information or advice you have provided to a patient? | 1. Always 2. Sometimes 3. Rarely 4. Never |
| 16. | Have you ever practiced a computerized nursing care documentation system currently in your hospital? | 1. Always 2. Sometimes 3. Rarely 4. Never |
| 17. | How often did you voluntarily report any medical error that occurs while providing patient care? | 1. Always 2. Sometimes 3. Rarely 4. Never |
| 18. | How often did you document the patient's response to the care you provide? | 1. Always 2. Sometimes 3. Rarely 4. Never |
