# Supplementary material for: Nursing patient record practice and associated factors among nurses working in North Shewa Zone public hospitals, Ethiopia
Source: Front Health Serv. 2024 Feb 8;4:1340252. doi: 10.3389/frhs.2024.1340252 (PMC10883157; doi:10.3389/frhs.2024.1340252)
Supplement: Supplementary file 2 [file Table2.docx]

**Informed consent form**

I have been informed that the interview is to gather information regarding the Nursing patient record management practice and associated factors among nurses working in North Shewa Zone Public Hospitals, Ethiopia. The objective, procedure, benefits, and harms of the study have been read and explained to me in a language I understand. I further appreciate that taking part in this study and withdrawing from participating at any time without having a reason is purely voluntary. I also agreed about the confidentiality of the responses to be at a higher possible level. Therefore; I declare my voluntary consent to participate in this study with my initials (signature) as indicated below.

Participant’s signature _______________ Date _________________

Data collector: Name__________________

Signature ______________ Date________________

Supervisor: Name: ____________________Signature: ____________ Date___________

If you have any questions, you can contact the following persons:

1. Mesfin Tadese Tel: +251 915839921 Email: [mesitad031@gmail.com](mailto:mesitad031@gmail.com)
2. Agizew Endale Tel: +251909668099 Email: [agizewendale2018@gmail.com](mailto:agizewendale2018@gmail.com)
3. Wondwosen Asegidew Tel: +251913046448 Email: [wondeasgw@gmail.com](mailto:wondeasgw@gmail.com)
4. Saba Desta Tel: +251911116065 Email: [sabadesta127@gmail.com](mailto:sabadesta127@gmail.com)
5. Wondimeneh Shibabaw Tel: +251912012516 Email: [wshibabaw2015@gmail.com](mailto:wshibabaw2015@gmail.com)

**Thank you for your cooperation!**
